# A comparative approach to stabilizing mechanisms between discrete- and continuous-time consumer-resource models

**DOI:** 10.1371/journal.pone.0265825

**Published:** 2022-04-12

**Authors:** Abhyudai Singh

**Affiliations:** Department of Electrical and Computer Engineering, Department of Biomedical Engineering, Department of Mathematical Sciences, Center for Bioinformatics and Computational Biology, University of Delaware, Newark, DE, United States of America; Universidade Federal de Lavras, BRAZIL

## Abstract

There is rich literature on using continuous-time and discrete-time models for studying population dynamics of consumer-resource interactions. A key focus of this contribution is to systematically compare between the two modeling formalisms the stabilizing/destabilizing impacts of diverse ecological processes that result in a density-dependent consumer attack rate. Inspired by the Nicholson-Bailey/Lotka-Volterra models in discrete-time/continuous-time, respectively, we consider host-parasitoid interactions with an arbitrary parasitoid attack rate that is a function of both the host/parasitoid population densities. Our analysis shows that a Type II functional response is stabilizing in both modeling frameworks only when combined with other mechanisms, such as mutual interference between parasitoids. A Type III functional response is by itself stabilizing, but the extent of attack-rate acceleration needed is much higher in the discrete-time framework, and its stability regime expands with increasing host reproduction. Finally, our results show that while mutual parasitoid interference can stabilize population dynamics, cooperation between parasitoids to handle hosts is destabilizing in both frameworks. In summary, our comparative analysis systematically characterizes diverse ecological processes driving stable population dynamics in discrete-time and continuous-time consumer-resource models.

## I. Introduction

Interaction between a resource (such as, a prey or host) and a consumer (such as, a predator or parasitoid) forms a core motif in ecological networks. Population dynamics of consumer-resource interactions has been extensively studied using two different approaches: continuous-time and discrete-time models. The continuous-time framework is generally used to model populations with overlapping generations and all year-round reproduction. In contrast, discrete-time models are more suited for populations with non-overlapping generations that reproduce in a discrete pulse determined by season. Perhaps, the classical example of this in continuous time is the Lotka-Volterra model

$$\frac{dh(t)}{dt}=rh\left(t\right)-ch\left(t\right)p\left(t\right)$$ (1a)

$$\frac{dp(t)}{dt}=ch\left(t\right)p\left(t\right)-\gamma p\left(t\right)$$ (1b)

[1–10]. Here *h*(*t*) and *p*(*t*) denote the population densities of the host and the parasitoid at time *t*, respectively. Parasitoids attack hosts with rate *c* with each parasitized host developing into a new parasitoid. Finally, 1/*γ* is the average lifespan of an individual parasitoid. It is well-known that the steady-state equilibrium of the Lotka-Volterra model is neutrally stable resulting in cycling population densities with a period of $\approx 2\pi /\sqrt{r\gamma }$ [1].

The analogous counterpart of the Lotka-Volterra model in discrete-time is the Nicholson-Bailey model

$$H_{t+1}=RH_{t}\text{exp}\left(-cP_{t}\right)$$ (2a)

$$P_{t+1}=RH_{t}\left[1-\text{exp}\left(-cP_{t}\right)\right]$$ (2b)

where we now use capital letters *H*_*t*_ and *P*_*t*_ to denote the adult host, and the adult parasitoid densities, respectively, in year *t* [4, 11–16]. The model reflects the synchronized annual life cycles of these insects living in the temperate regions of the world [17–20]. More specifically, if *R* > 1 denotes the number of viable eggs produced per adult host, then *RH*_*t*_ is the host larval density that becomes exposed to parasitoid attacks. Parasitoids attack and parasitize hosts at a constant rate *c*, resulting in the fraction exp(−*cP*_*t*_) that escapes parasitism to become the adult hosts for the next year. Similarly, the fraction 1 − exp(−*cP*_*t*_) of parasitized larvae give rise to adult parasitoids in the next generation. The Nicholson-Bailey model is characterized by diverging oscillations in population densities resulting in an unstable population dynamics [11].

The neutrally stable Lokta-Volterra equilibrium and the unstable Nicholson-Bailey equilibrium catalyzed rich theoretical work in understanding how individual ecological mechanisms (such as a Type II functional response, interference between parasitoid in attacking hosts, etc.) promote stability of host-parasitoid interactions, especially in the context of biological control of pest species [1, 21–24]. A key contribution of this work is to study the combined effect of different forms of density-dependence in the parasitoid attack rate and quantify their overall stabilizing effect in a single stability criterion. This gives us a holistic picture of how a mixture of processes impact population dynamics. For example, while a Type II functional response is destabilizing in both the continuous- and discrete-time frameworks, combining it with some form of parasitoid interference can lead to stable population dynamics. Another novelty of this work is the comparison of stability regimes between the modeling formalisms, and how these stability regimes change with relevant ecological parameters, such as the extent of host reproduction. This comparison is specifically important to elucidate the general forms of density-dependence in the parasitoid attack rate that stabilizes population dynamics irrespective of the modeling framework, and hence are robust to model choice. Such general features are particularly relevant for actual populations that may not exactly follow the idealistic modeling assumption of either Lotka-Volterra or Nicholson-Bailey formalisms and may be best described by an intermediate hybrid modeling framework.

**Fig 1:**
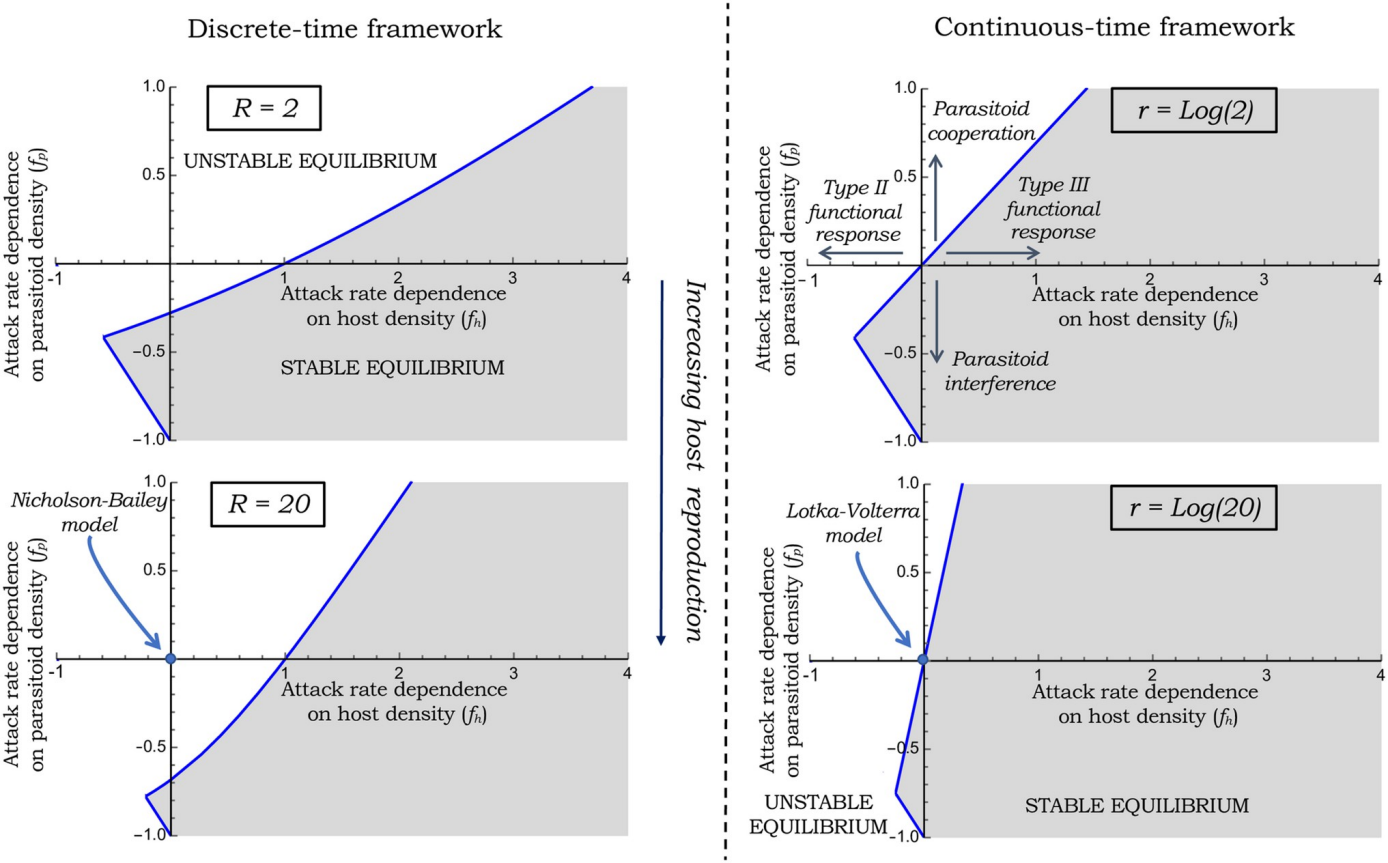
The stability region (grey shaded area) as determined by (5) and (10) in continuous time (right) and discrete-time (left), respectively, is plotted as function of *f*_*h*_ (attack-rate sensitivity to the host density) and *f*_*p*_ (attack-rate sensitivity to the parasitoid density). The origin *f*_*h*_ = *f*_*p*_ = 0 represents the classical Lotka-Volterra and Nicholson-Bailey models. While the former model in continuous time is on the edge of stability indicating a neutrally-stable equilibrium, the Nicholson-Bailey model in discrete time is outside the grey shaded area indicating an unstable equilibrium. The stability regions are plotted for two different levels of host reproduction—*R* = 2 (top) and *R* = 20 (bottom) with *r* = log *R* and *γ* = 1.

The manuscript is organized as follows: in Section II we formulate the generalized Lotka-Volterra model and derive criteria for stable population dynamics. The same process is repeated in Section III for an analogously formulated Nicholson-Bailey model. We compare stability regions in Section IV summarizing the findings in Fig 1, and put these results in the context of known literature and highlight new insights arising from this study.

## II. Stability condition for a generalized Lotka-Volterra model

Consider a generalized Lotka-Volterra model

$$\frac{dh(t)}{dt}=rh\left(t\right)-f\left(h,p\right)h\left(t\right)p\left(t\right)$$ (3a)

$$\frac{dp(t)}{dt}=f\left(h,p\right)h\left(t\right)p\left(t\right)-\gamma p\left(t\right)$$ (3b)

with an attack rate *f*(*h*, *p*) that is an arbitrary continuously-differentiable function of both the host and parasitoid population densities. Such an attack rate captures diverse ecological processes:

- Function *f* decreasing with host density corresponds to a Type II functional response. In contrast, *f* varies non-monotonically with host density for a Type III response with the attack rate increasing (accelerating) with host density at low densities and then decreasing at high host densities. This non-monotonicity results in the classical sigmoidal shape of net attack rate *f*(*h*, *p*)*h*(*t*) per parasitoid [25–35].
- Function *f* decreasing (increasing) with parasitoid density corresponds to mutual interference (cooperation) between parasitoids [36–41].

We assume that model (3) has a unique non-trivial equilibrium given as the solution to

$$\begin{array}{c} f\left(h^{*},p^{*}\right)=\frac{r}{p^{*}},\;\;p^{*}=\frac{rh^{*}}{\gamma }. \end{array}$$ (4)

where h* and p* represent the equilibrium host and parasitoid densities, respectively. Note that the model also has a trivial equilibrium h* = p* = 0, but we are primarily interested in the properties of the non-trivial equilibrium. In Appendix A, we provide explicit conditions for a wide range of attack rates *f* that result in a unique non-trivial equilibrium.

Our analysis in Appendix B shows that this equilibrium is asymptotically stable, if and only if,

$$\begin{array}{c} f_{p}<\frac{rf_{h}}{\gamma },\;\;1+f_{h}+f_{p}>0, \end{array}$$ (5)

where *f*_*p*_ and *f*_*h*_

$$f_{h}≔\frac{h^{*}}{f(h^{*},p^{*})}\frac{\partial f(h,p)}{\partial h}{|}_{h=h^{*},p=p^{*}},$$ (6a)

$$f_{p}≔\frac{p^{*}}{f(h^{*},p^{*})}\frac{\partial f(h,p)}{\partial p}{|}_{h=h^{*},p=p^{*}},$$ (6b)

are the dimensionless log sensitivities of the attack rate to the host and parasitoid densities, respectively. It is reasonable to have the net attack rate per host *f*(*h*, *p*)*p* be a non-decreasing function of the parasitoid density that constrains *f*_*p*_ ≥ −1. Similarly, we constrain *f*_*h*_ ≥ −1 that ensures the net attack rate per parasitoid *f*(*h*, *p*)*h* be a non-decreasing function of the host density. If any one of the inequalities in (5) does not hold, then the equilibrium is locally unstable and this instability often manifests in a stable limit cycle [42, 43].

## III. Stability conditions for a generalized Nicholson-Bailey model

We next consider a potential discrete-time counterpart of the generalized Lotka-Volterra model. One phenomenological approach to obtain this model is to simply substitute *c* in (2) with *f*(*H*_*t*_, *P*_*t*_) yielding

$$H_{t+1}=RH_{t}\;\text{exp}\left(-f\left(H_{t},P_{t}\right)P_{t}\right)$$ (7a)

$$P_{t+1}=RH_{t}\left[1-\text{exp}\left(-f\left(H_{t},P_{t}\right)P_{t}\right)\right]$$ (7b)

[44]. Note that during the time window when hosts are vulnerable to parasitoid attacks, the density of unparasitized host continuously decreases. To capture such effects of continuously changing populations, a semi-discrete or hybrid formalism has been proposed to mechanistically formulate the corresponding discrete-time model [45–51]. Briefly, an ordinary-differential equation model describing population interaction during the attack time window is solved to derive the update functions of the discrete-time model. Importantly, this mechanistic approach can yield contrasting results from the phenomenological approach—a Type III functional response is stabilizing in the semi-discrete approach [48, 52], but always destabilizing if one considers (7) with an analogous attack rate [48].

Referring the reader to Appendix C for details, the semi-discrete approach yields the following model in the discrete-time formalism that is analogous to the continuous-time counterpart (3)

$$H_{t+1}=\frac{RH_{t}}{{\left(1+f_{h}{\left(RH_{t}\right)}^{f_{h}}P_{t}^{1+f_{p}}\right)}^{\frac{1}{f_{h}}}}$$ (8a)

$$P_{t+1}=RH_{t}\left(1-\frac{1}{{\left(1+f_{h}{\left(RH_{t}\right)}^{f_{h}}P_{t}^{1+f_{p}}\right)}^{\frac{1}{f_{h}}}}\right).$$ (8b)

Recall from (6) that *f*_*h*_ and *f*_*p*_ are the log sensitivities of the parasitoid attack rate to the host and parasitoid densities, respectively. This model has a unique non-trivial equilibrium

$$\begin{array}{c} H^{*}={\left(\frac{1-R^{-f_{h}}}{f_{h}{\left(R-1\right)}^{1+f_{p}}}\right)}^{\frac{1}{1+f_{h}+f_{p}}},\;P^{*}=\left(R-1\right)H^{*}, \end{array}$$ (9)

which is asymptotically stable, if and only if,

$$\begin{array}{c} f_{p}<\frac{R+\left(f_{h}\left(R-1\right)-R\right)R^{f_{h}}}{R\left(R^{f_{h}}-1\right)},\;\;1+f_{h}+f_{p}>0. \end{array}$$ (10)

Note that the second inequality is the same as in (5), and hence 1 + *f*_*h*_ + *f*_*p*_ > 0 is a *necessary* condition for stability in both modeling frameworks. One can consider small and large values of *R* for which the stability condition reduces to

$$f_{p}<0,\;\;1+f_{h}+f_{p}>0,\;\text{when}\;R\to 1,$$ (11a)

$$f_{p}<f_{h}-1,\;\;1+f_{h}+f_{p}>0,\;\text{when}\;R\to \infty ,$$ (11b)

respectively.

## IV. Comparison of stability regimes

To understand the stabilizing/destabilizing effects of diverse ecological processes we plot the stability regions as predicted by inequalities (5) and (10) in Fig 1. For an analogous comparison, the hosts growth rate *r* in the continuous-time framework is related to *R* in the discrete-time framework by *r* = log(*R*). In Fig 1, *f*_*h*_ = *f*_*p*_ = 0 represents the classical Lotka-Volterra/Nicholson-Bailey models and

- Going right along the positive x-axis (*f*_*h*_ > 0) corresponds to an attack rate that accelerates with increasing host density as in a Type III functional response.
- Going left along the negative x-axis (*f*_*h*_ < 0) corresponds to an attack rate that decreases with increasing host density as in a Type II functional response.
- Going up along the positive y-axis (*f*_*p*_ > 0) corresponds to an attack rate that increases with parasitoid density capturing cooperation between parasitoids to handle hosts.
- Going down along the negative y-axis (*f*_*p*_ < 0) corresponds to an attack rate that decreases with increasing parasitoid density corresponding to mutual interference between parasitoid, or aggregation of parasitoid attacks to a subpopulation of high-risk hosts [22, 48, 53–60].

A straightforward observation from Fig 1 is that the region of stability is larger in continuous time as compared with discrete time, but in some limits, they turn out to be identical.

### A. Comparison of stability conditions with respect to host reproduction

An important result that emerges from the inequalities (5) and (10) is that for low levels of host reproduction (i.e., *R* → 1 and *r* → 0), both modeling frameworks have exactly the same stability criterion

$$\begin{array}{c} f_{p}<0,1+f_{h}+f_{p}>0 \end{array}$$ (12)

showing that an attack rate decreasing with increasing parasitoid density is a *necessary* condition for stability. For high levels of host reproduction (i.e., *R* → ∞ and *r* → ∞), *f*_*h*_ > 0 becomes the *necessary and sufficient* condition for stability in the Lotka-Volterra framework. In the Nicholson-Bailey framework, the stability condition for *R* → ∞ is given by (11b) that corresponds to the two lines meeting at *f*_*h*_ = 0 and *f*_*p*_ = −1. This implies that *f*_*h*_ > 0 is only a *necessary* condition for stability in the discrete-time framework for large values of *R* and one further requires *f*_*p*_ < *f*_*h*_ − 1 for stability. It is important to point that the limit *R* → ∞ is less ecologically relevant, as for most natural systems *R* is expected to be less than 10 [1].

### B. Impact of functional responses

We first consider the effect of a Type II functional response

$$\begin{array}{c} f\left(h\right)=\frac{c_{1}}{1+c_{1}T_{h}h}, \end{array}$$ (13)

where *c*_1_ is the attack rate at low-host density and *T*_*h*_ is the parasitoid handling time. In this case, the dimensionless log sensitivities of the attack rate to the host density

$$\begin{array}{c} f_{h}=-\frac{c_{1}T_{h}h}{1+c_{1}T_{h}h} \end{array}$$ (14)

is negative, and *f*_*h*_ approaches −1 as handling times becomes longer and longer. In the context of Fig 1, this corresponds to the neutrally stable Lotka-Volterra equilibrium moving left on the negative x-axis, and this destabilizing effect of handling times is consistent with known literature [61].

Interestingly, a Type II functional response (*f*_*h*_ < 0) can provide stability if combined with other mechanisms, such as, interference in parasitoid attack where *f*_*p*_ < 0 (grey-shaded area in the third quadrant). Note that this grey-shaded area decreases in size with increasing reproduction, suggesting that the stability arising from the mixture of handling times and parasitoid interference is more likely to operate at low host proliferation. The meeting point of two lines in the third quadrant gives the necessary condition for stability

$$f_{h}>-\frac{1}{1+\frac{r}{\gamma }}\;\text{in}\;\text{continuous}\;\text{time}$$ (15a)

$$f_{h}>\frac{\text{log}R-\text{log}\left(2R-1\right)}{\text{log}R}\;\text{in}\;\text{discrete}\;\text{time}$$ (15b)

that reduces to *f*_*h*_ > −1 in the limit *R* → 1 & *r* → 0. These limits on *f*_*h*_ have important implications suggesting that long handling times that cause *f*_*h*_ → −1 can drive instability even in the presence of parasitoid interference in both modeling frameworks.

A Type III functional response (*f*_*h*_ > 0 and *f*_*p*_ = 0) is stabilizing in both framework but the degree of attack-rate acceleration needed for stability is much higher in the discrete-time framework: *f*_*h*_ > 1 in the discrete-time framework compared to *f*_*h*_ > 0 in the continuous-time framework.

Recall that one of the inequalities needed for stability 1 + *f*_*h*_ + *f*_*p*_ > 0 is the same for both frameworks. The other inequality in the continuous-time framework is anchored at the origin and rotates anticlockwise with increasing *R*. In contrast, the other inequality in the discrete-time framework is anchored at *f*_*h*_ = 1, *f*_*h*_ = 0 and also rotates anticlockwise with increasing *R*. This leads to the stability region related to a Type II functional response shrinking with increasing *R*. However, the stability region related to Type III responses expands with increasing *R* in the continuous-time framework. The non-origin anchoring in the discrete-time framework leads to the stability region related to Type III responses expanding with increasing *R* for *f*_*h*_ > 1, but shrinking for 0 < *f*_*h*_ < 1.

### C. Impact of a parasitoid-dependent attack rate

For a parasitoid-dependent attack rate (*f*_*h*_ = 0) one can see that *f*_*p*_ > 0 (i.e., cooperation between parasitoids) is not stabilizing in both frameworks. In contrast, mutual interference between parasitoids is stabilizing with the stability criterion reducing to

$$f_{p}<0\;\;\text{in}\;\text{continuous}\;\text{time}$$ (16a)

$$f_{p}<-\frac{R\text{ln}\left(R\right)+1-R}{R\text{ln}\left(R\right)}\;\text{in}\;\text{discrete}\;\text{time}.$$ (16b)

An interesting observation from the fourth-quadrant of the discrete-time stability region is that while values of

$$\begin{array}{c} 0>f_{p}>-\frac{R\text{ln}\left(R\right)+1-R}{R\text{ln}\left(R\right)} \end{array}$$ (17)

and

$$\begin{array}{c} 0<f_{h}<1 \end{array}$$ (18)

are by themselves not stabilizing, their combination can lead to stability. Thus, moderate levels of parasitoid interference together with attack-rate acceleration to host density can stabilize population dynamics in the Nicholson-Bailey formulation of host-parasitoid population dynamics. Finally, we point out that the stability resulting from a combination of cooperation between parasitoids and a Type III functional response (grey-shaded region in the first quadrant), but increasing parasitoid cooperation also requires a higher degree of attack-rate acceleration to keep the system dynamics stable.

## V. Conclusion

In summary, this contribution combined the stabilizing effects of diverse ecological processes into a single stability criterion in both the Lotka-Volterra ad Nicholson-Bailey modeling formalisms. These results highlight general features that are stabilizing, irrespective of model choice. For example, while destabilizing effects of Type II functional response are well known in the literature, combining it with parasitoid interference can lead to stability (the third quadrant in Fig 1), and stability arising from this mixture is more likely to be prevalent for low values of *R* and *r*. Eq (16) quantifies the limits to such a strategy and suggests that long handling times cannot be stabilized irrespective of parasitoid interference.

Recent fieldwork has documented cooperation between parasitoids, where groups of female *Sclerodermus harmandi* wasps are shown to be more successful in exploiting large-sized host individuals as compared to individual wasps [40]. Our analysis shows that such cooperation between parasitoids can have a destabilizing effect, but this can be countered by the stabilizing effect of a Type III functional response. Indeed, given cooperation between parasitoids, a Type III response is necessary for stability. For high values of *R* and *r*, stability is much more likely to arise from a Type III response with either parasitoid interference or cooperation.

It is perhaps intuitive that the overall stability regime is larger in the continuous-time framework, but interestingly, our analysis reveals that the stability region is identical in the limit of low host reproduction. An important restriction of this work is that there is no limit to host growth in the absence of the parasitoid, and these results could be further generalized to consider a host carrying capacity, which can be stabilizing in both frameworks [1, 46]. Another interesting direction of future work would be to expand these stability criteria in the context of two parasitoid species attacking the same host, or two different host species attacked by a common parasitoid providing a holistic impact of diverse mechanisms on the stability of complex consumer-resource models.

## Appendix A: Uniqueness of non-trivial equilibrium in the generalized Lotka-Volterra model

A functional response in the parasitoid attack rate can be modeled using a function *f* that only depends on the host density

$$\begin{array}{c} f\left(h\right)=\frac{c_{1}h^{q}}{1+c_{1}T_{h}h^{q+1}} \end{array}$$ (19)

with parameter *c*_1_ > 0, *T*_*h*_ is the parasitoid handling time, *q* = 0 corresponds to a Type II functional response, and *q* > 0 captures a Type III functional response. The non-trivial equilibrium is given as the solution to (4), which can be rewritten as

$$\begin{array}{c} h^{*}f\left(h^{*}\right)=\gamma \;\;p^{*}=\frac{rh^{*}}{\gamma }. \end{array}$$ (20)

Substituting (19) in (20), h* is the solution to

$$\begin{array}{c} \frac{c_{1}{h^{*}}^{q+1}}{1+c_{1}T_{h}{h^{*}}^{q+1}}=\gamma \end{array}$$ (21)

Assuming the parasitoid handling time is smaller that the average parasitoid lifespan (i.e., *T*_*h*_ < 1/*γ*), (21) will have a unique solution as the left-hand-side of (21) is a monotonically increasing function that starts at zero and saturates at 1/*T*_*h*_ > *γ*, and hence, h* *f*(h*) will intersect with *γ* only once. Having obtained the unique h*, the corresponding parasitoid density is given by $p^{*}=\frac{rh^{*}}{\gamma }$.

Let us consider a parasitoid-dependent attack rate

$$\begin{array}{c} f\left(p\right)=c_{1}p^{\alpha } \end{array}$$ (22)

where *c*_1_ > 0 and −1 < *α* < 1, with negative (positive) values of *α* denoting parasitoid interference (cooperation). In this case the unique non-trivial equilibrium will be

$$\begin{array}{c} p^{*}={\left(\frac{r}{c_{1}}\right)}^{\frac{1}{1+\alpha }},\;\;h^{*}=\frac{p^{*}\gamma }{r}. \end{array}$$ (23)

## Appendix B: Stability criterion for the generalized Lotka-Volterra model

Linearizing the right-hand-side of (3) around the equilibrium yields the following Jacobian matrix

$$\begin{array}{c} A=[\begin{array}{cc} -rf_{h} & -f_{p}\gamma -\gamma \\ rf_{h}+r & \gamma f_{p} \end{array},] \end{array}$$ (24)

and stability requires a Hurwitz matrix whose eigenvalues have negative real parts [62, 63]. For a two-dimensional system, the equilibrium is asymptotically stable, if and only if, the determinant of the *A* matrix is positive and its trace is negative [62, 63]. This implies that the equilibrium obtained as the solution to (4) is asymptotically stable, if and only if, both inequalities in (5) hold.

## Appendix C: Stability criterion for the generalized Nicholson-Bailey model

The semi-discrete approach models the host-parasitoid interaction during the host’s vulnerable stage as an ordinary differential equation. Let *τ* denote the time within the host vulnerable stage that varies from 0 to *T* corresponding to the start and end of the vulnerable stage. The densities of parasitoids, un-parasitized and parasitized host larvae at time *τ* within the vulnerable stage of year *t* are represented by *P*(*τ*, *t*), *L*(*τ*, *t*), *I*(*τ*, *t*), respectively. These densities evolve as per the dynamical system

$$\frac{dP\left(\tau ,t\right)}{d\tau }=-\gamma_{P}P\left(\tau ,t\right)$$ (25a)

$$\frac{dL\left(\tau ,t\right)}{d\tau }=-cP\left(\tau ,t\right)L\left(\tau ,t\right)-\gamma_{L}L\left(\tau ,t\right)$$ (25b)

$$\frac{dI\left(\tau ,t\right)}{d\tau }=cP\left(\tau ,t\right)L\left(\tau ,t\right)-\gamma_{I}I\left(\tau ,t\right),$$ (25c)

where *c* represents the parasitoid’s attack rate *per host*, and *γ*_*P*_, *γ*_*L*_, *γ*_*I*_ are the death rates of the respective species. Assuming *P*_*t*_ parasitoids, *RH*_*t*_ host larvae, and no parasitized larvae at the start of the vulnerable period (*τ* = 0), solving the above differential equations with initial conditions

$$\begin{array}{c} L\left(0,t\right)=RH_{t},\;\;P\left(0,t\right)=P_{t},\;\;I\left(0,t\right)=0 \end{array}$$ (26)

predicts the parasitized and unparasitized larval populations at the end of the season (*τ* = *T*). This leads to a more general discrete-time model

$$H_{t+1}=F\left(H_{t},P_{t}\right)$$ (27a)

$$P_{t+1}=G\left(H_{t},P_{t}\right)$$ (27b)

where update functions are obtained by setting

$$F\left(H_{t},P_{t}\right)=L\left(T,t\right)$$ (28a)

$$G\left(H_{t},P_{t}\right)=I\left(T,t\right).$$ (28b)

Solving (25) for a constant attack rate *c* with no mortalities (*γ*_*P*_ = *γ*_*L*_ = *γ*_*I*_ = 0), and assuming *T* = 1 without loss of any generality, yields the Nicholson-Bailey model (2).

To capture a generalized parasitoid attack with log sensitivities *f*_*h*_ (with respect to the host) and *f*_*p*_ (with respect to the parasitoid), we replace *c* in (25) with the monomial $L{\left(\tau ,t\right)}^{f_{h}}P_{t}^{f_{p}}$. In the absence of any mortalities (*γ*_*P*_ = *γ*_*L*_ = *γ*_*I*_ = 0) and again assuming *T* = 1, the above semi-discrete approach results in the model (8). Linearizing the right-hand-side of (8) around the equilibrium (9) results in the Jacobian matrix

$$\begin{array}{c} A=[\begin{array}{cc} 1-f_{h}{H^{*}}^{f_{h}}{P^{*}}^{1+f_{p}} & -\left(1+f_{p}\right){H^{*}}^{1+f_{h}}{P^{*}}^{f_{p}}\\ R-1+f_{h}{H^{*}}^{f_{h}}{P^{*}}^{1+f_{p}} & \left(1+f_{p}\right){H^{*}}^{1+f_{h}}{P^{*}}^{f_{p}} \end{array}], \end{array}$$ (29)

and stability in the discrete-time formalism requires all eigenvalues of *A* to have an absolute value less than one [63, 64]. For a 2 × 2 matrix, the stability criterion can be written in terms of the determinant and the trace of *A*—the equilibrium (9) is stable, if and only if,

$$\begin{array}{c} 1-Det\left(A\right)>0 \end{array}$$ (30)

$$\begin{array}{c} 1+Tr\left(A\right)-Det\left(A\right)>0 \end{array}$$ (31)

$$\begin{array}{c} 1+Tr\left(A\right)+Det\left(A\right)>0. \end{array}$$ (32)

It turns out that the last inequality always holds, and the first two inequalities yield the stability conditions (10).
